# Clinical characteristics of lurasidone-treated patients in Spain using Natural Language Processing – A real-world data study with Electronic Health Records

**DOI:** 10.1192/j.eurpsy.2022.544

**Published:** 2022-09-01

**Authors:** C. De La Pinta, I. Gabarda

**Affiliations:** 1 Medsavana, Medcore, Madrid, Spain; 2 Angelini Pharma España, S.L.U., Medical Department, Barcelona, Spain

**Keywords:** schizophrénia, Electronic Health Records, lurasidone, Natural Language Processing

## Abstract

**Introduction:**

Schizophrenia is a chronic neuropsychiatric disorder which affects over 20 million people worldwide. Atypical antipsychotics are the first-line choice for the treatment of schizophrenia due to improved tolerability and diminished risk of extrapyramidal symptoms. Lurasidone is an atypical antipsychotic approved in Spain for the treatment of schizophrenia in September 2019. An RWD-based picture of lurasidone use is necessary to better understand its impact in routine clinical practice.

**Objectives:**

To set up a methodology based on Natural Language Processing (NLP) and machine learning for the analysis of the free-text information contained in the EHRs of patients treated with lurasidone in Spain.

**Methods:**

A multicenter, retrospective study based on RWD collected in EHRs of lurasidone users will be conducted in hospitals from the Spanish National Healthcare System. Information extracted from the free text in EHRs using NLP will be treated and analyzed as big data.

**Results:**

A study database for lurasidone-treated patients in Spain has been instituted using the EHRead® technology (* Figure 1*), which applies machine learning and deep learning to extract, analyze, and interpret the free-text information written in their de-identified EHRs. Sociodemographic and clinical variables in EHRs from September 2019 until the most recent data available are being collected to describe the target patient population and address treatment-related outcomes.

**Figure:**
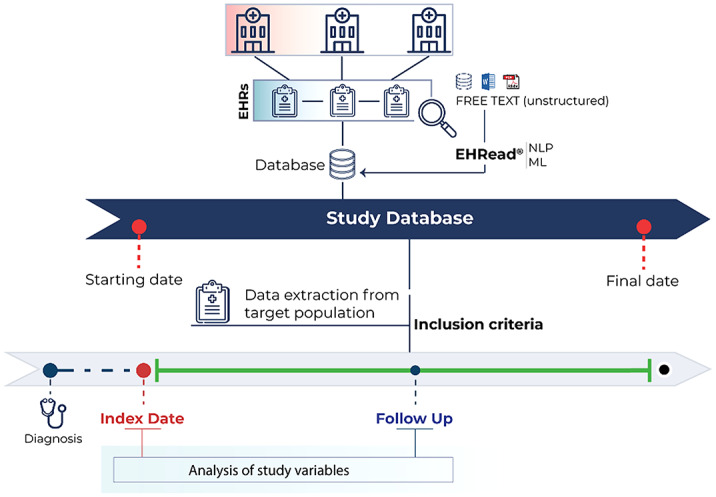

**Conclusions:**

NLP of free text in EHRs of lurasidone-treated patients renders a real-world picture of lurasidone usage in Spain. Studies using artificial intelligence techniques represent a novel source of information regarding psychiatric disorders and their clinical management.

**Disclosure:**

I. Gabarda is employee at Angelini Pharma España, S.L.U. and C. de la Pinta is employee at Medsavana.

